# 3-(4-Chloro­phen­yl)-5-phenyl-4,5-di­hydro-1,3-oxazole

**DOI:** 10.1107/S1600536812043711

**Published:** 2012-10-27

**Authors:** Arun M. Islor, Rajiv Yaradoni, B. Garudachari, Thomas Gerber, Eric Hosten, Richard Betz

**Affiliations:** a National Institute of Technology-Karnataka, Department of Chemistry, Medicinal Chemistry Laboratory, Surathkal, Mangalore 575 025, India; b Nelson Mandela Metropolitan University, Summerstrand Campus, Department of Chemistry, University Way, Summerstrand, PO Box 77000, Port Elizabeth, 6031, South Africa

## Abstract

In the title compound, C_15_H_12_ClNO, the isoxazoline ring adopts an envelope conformation with the C atom bearing an unsubstituted phenyl ring as the flap atom. The chlorinated phenyl group is nearly in-plane with the four coplanar atoms of the heterocycle and the corresponding mean planes enclosing an angle of 1.16 (7)°. The unsubstituted phenyl group attached to the envelope flap atom approaches a nearly perpendicular orientation relative to the isoxazoline ring with a dihedral angle of 74.93 (7)°. In the crystal, weak C—H⋯O, C—H⋯N and C—H⋯π inter­actions connect the mol­ecules into layers perpendicular to the *a* axis.

## Related literature

For the biological and medicinal importance of isoxazole compounds, see: Miller *et al.* (2009 ▶); Prasad *et al.* (2007 ▶). For their use in ring-opening polymerizations, see: Wiesbrock *et al.* (2005 ▶). For the puckering analysis of five-membered rings, see: Cremer & Pople (1975 ▶). For graph-set analysis of hydrogen bonds, see: Etter *et al.* (1990 ▶); Bernstein *et al.* (1995 ▶).
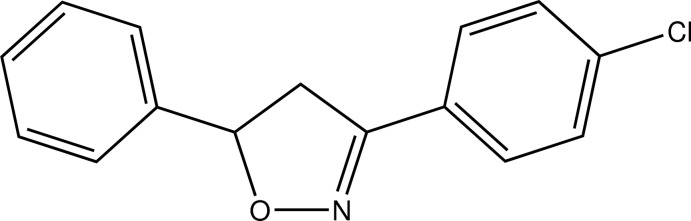


## Experimental

### 

#### Crystal data

C_15_H_12_ClNO
*M* *_r_* = 257.71
Monoclinic, 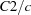

*a* = 29.797 (5) Å
*b* = 10.717 (5) Å
*c* = 8.086 (5) Å
β = 103.088 (5)°
*V* = 2515 (2) Å^3^
*Z* = 8
Mo *K*α radiation
μ = 0.29 mm^−1^
*T* = 200 K
0.58 × 0.42 × 0.21 mm

#### Data collection

Bruker APEXII CCD diffractometer
Absorption correction: multi-scan (*SADABS*; Bruker, 2008 ▶) *T*_min_ = 0.850, *T*_max_ = 0.943
11843 measured reflections
3132 independent reflections
2637 reflections with *I* > 2σ(*I*)
*R*_int_ = 0.014

#### Refinement

*R*[*F* ^2^ > 2σ(*F* ^2^)] = 0.037
*wR*(*F* ^2^) = 0.103
*S* = 1.03
3132 reflections
163 parameters
H-atom parameters constrained
Δρ_max_ = 0.31 e Å^−3^
Δρ_min_ = −0.23 e Å^−3^

### 

Data collection: *APEX2* (Bruker, 2010 ▶); cell refinement: *SAINT* (Bruker, 2010 ▶); data reduction: *SAINT*; program(s) used to solve structure: *SHELXS97* (Sheldrick, 2008 ▶); program(s) used to refine structure: *SHELXL97* (Sheldrick, 2008 ▶); molecular graphics: *ORTEP-3* (Farrugia, 1997 ▶) and *Mercury* (Macrae *et al.*, 2008 ▶); software used to prepare material for publication: *SHELXL97* and *PLATON* (Spek, 2009 ▶).

## Supplementary Material

Crystal structure: contains datablock(s) I, global. DOI: 10.1107/S1600536812043711/gk2526sup1.cif

Supplementary material file. DOI: 10.1107/S1600536812043711/gk2526Isup2.cdx

Structure factors: contains datablock(s) I. DOI: 10.1107/S1600536812043711/gk2526Isup3.hkl

Supplementary material file. DOI: 10.1107/S1600536812043711/gk2526Isup4.cml

Additional supplementary materials:  crystallographic information; 3D view; checkCIF report

## Figures and Tables

**Table 1:** Hydrogen-bond geometry (Å, °) *Cg* is the centroid of the C11–C16 ring.

*D*—H⋯*A*	*D*—H	H⋯*A*	*D*⋯*A*	*D*—H⋯*A*
C12—H12⋯N1^i^	0.95	2.74	3.657 (2)	163
C12—H12⋯O1^i^	0.95	2.65	3.390 (2)	135
C2—H2*B*⋯O1^ii^	0.99	2.67	3.466 (2)	138
C26—H26⋯O1^ii^	0.95	2.70	3.431 (2)	134
C22—H22⋯*Cg* ^iii^	0.95	2.81	3.721 (3)	162

Symmetry codes: (i) 
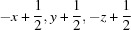
; (ii) 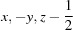; (iii) 
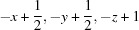
.

**Fig. 1.**
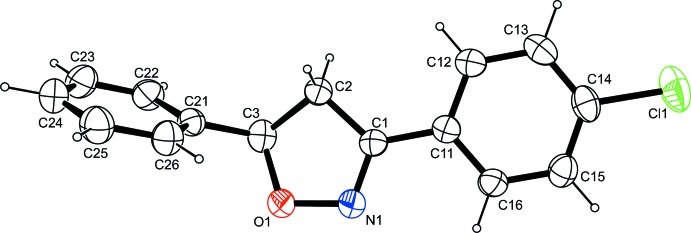
The molecular structure of the title compound, with anisotropic displacement ellipsoids drawn at the 50% probability level.

**Fig. 2.**
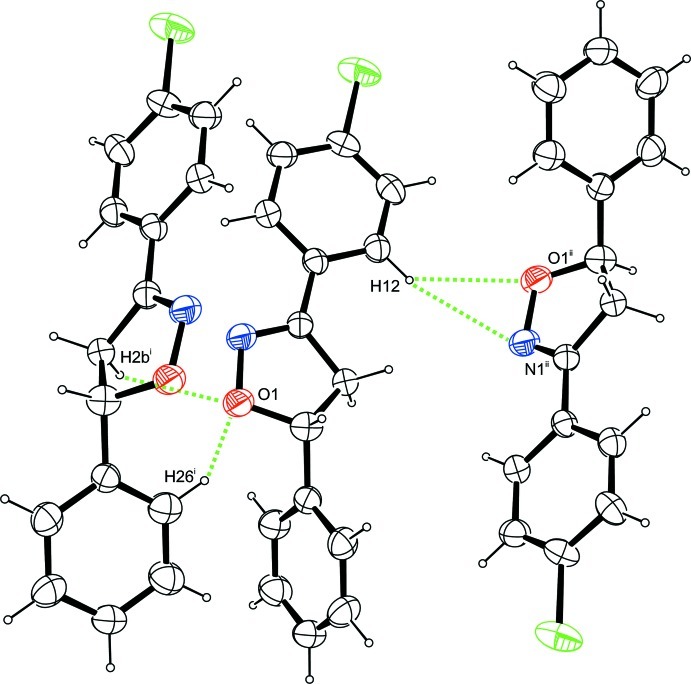
Intermolecular contacts, viewed approximately along [0 - 1 -1]. Symmetry operators: ix, -y, z + 1/2; ii -x + 1/2, y + 1/2, -z + 1/2.

**Fig. 3.**
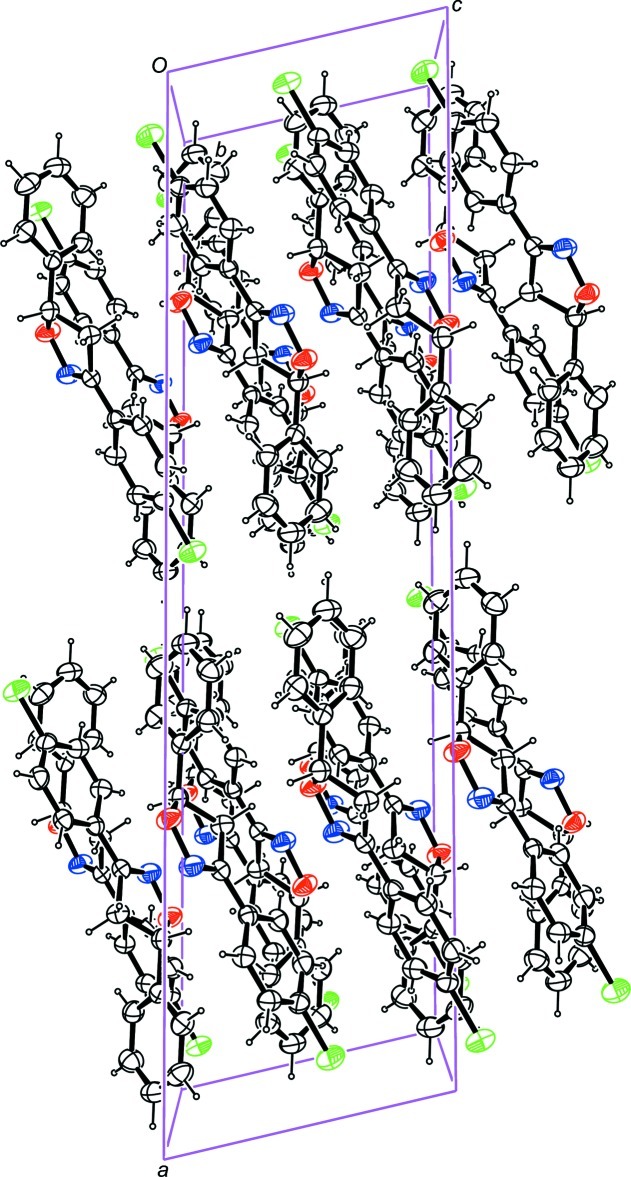
Molecular packing of the title compound, viewed along [0 1 0] (anisotropic displacement ellipsoids drawn at 50% probability level).

## supplementary crystallographic information

### Comment

Isoxazoles are well known organic compounds which are included in a variety of complex biologically active structures and play a role as catalyst, ligands and intermediates for functional compounds (Miller *et al.*, 2009; Prasad *et al.*, 2007). Isoxazoles appear in numerous medicinally active compounds and natural products of biological significance. Additionally, they are valuable as synthetic intermediates or protecting groups in organic synthesis. Also, isoxazoles serve as monomers for the synthesis of substituted poly(imine)s by cationic ring-opening polymerization (Wiesbrock *et al.*, 2005). Due to our interest in developing new isoxazole-based heterocycles, we have synthesized the title compound to study its crystal structure.

The title molecule features a chlorinated as well as a non-halogenated phenyl group as substituents on a central isoxazole core. The latter one adopts a ^5^*E* conformation with the flap atom on C3 (Cremer & Pople, 1975). While the halogenated phenyl group is nearly in-plane with the isoxazoline moiety – the least-squares planes defined by the respective intracyclic atoms intersect at an angle of 7.16 (7) ° only – the non-substituted phenyl group adopts a nearly perpendicular orientation towards the isoxazole moiety. The corresponding least-squares planes in the latter case enclose an angle of 74.93 (7) ° (Fig. 1).

In the crystal, only weak C–H···O and C–H···N contacts whose range falls slightly below the sum of van-der-Waals radii of the atoms participating in them are observed. The hydrogen atom that is part of the C–H···N contact stems from the chlorinated phenyl substituent and is also the origin of a bifuracated hydrogen bond that extends to the oxygen atom as acceptor. The C–H···O contacts are supported by the intracyclic methylene group as well as a hydrogen atom on the non-substituted phenyl group. Taking into account the latter two findings, the oxygen atom acts as threefold acceptor. Metrical parameters as well as information about the symmetry codes for these contacts are summarized in Table 1. In total, the molecules are connected to layers perpendicular to the crystallographic *a* axis. In terms of graph-set analysis (Etter *et al.*, 1990; Bernstein *et al.*, 1995), the descriptor for these contacts is *C*^1^_1_(4)*C*^1^_1_(5)*C*^1^_1_(5)*C*^1^_1_(6) on the unary level. The shortest intercentroid distance between two aromatic systems was measured at 4.709 (3) Å and is observed between the halogenated phenyl group and its symmetry-generated equivalent (Fig. 2).

The packing of the title compound in the crystal structure is shown in Figure 3.

### Experimental

An equimolar mixture of 1-(4-chlorophenyl)-*N*-hydroxymethanimine (0.5 g, 0.0032 mol), *N*-chloro succinamide (0.58 g, 0.0032 mol) and sodium bicarbonate (0.537 g, 0,0064 mol) in dichloromethane (10 ml) and water (10 ml) was stirred at 0 °C for 1 h. Styrene (0.366 g, 0.0035 mol) was then added to the reaction mixture and stirring was continued for another 12 h at room temperature. After completion of the reaction, the reaction mixture was concentrated and purified by column chromatography using petrol ether and ethyl acetate (*v*/*v* = 1:1) as the eluent to afford the title compound as a white solid, yield: 0.63 g (76.8%) (ChemSpider ID: 10496235).

### Refinement

Carbon-bound H atoms were placed in calculated positions (C—H 0.95 Å for aromatic carbon atoms, C—H 1.00 Å for methine groups and C—H 0.99 Å for methylene groups) and were included in the refinement in the riding model approximation, with *U*(H) set to 1.2*U*_eq_(C).

### Crystal data

**Table:**

C_15_H_12_ClNO	*F*(000) = 1072
*M**_r_* = 257.71	*D*_x_ = 1.361 Mg m^−^^3^
Monoclinic, *C*2/*c*	Melting point = 406–408 K
Hall symbol: -C 2yc	Mo *K*α radiation, λ = 0.71069 Å
*a* = 29.797 (5) Å	Cell parameters from 7100 reflections
*b* = 10.717 (5) Å	θ = 2.8–28.3°
*c* = 8.086 (5) Å	µ = 0.29 mm^−^^1^
β = 103.088 (5)°	*T* = 200 K
*V* = 2515 (2) Å^3^	Block, colourless
*Z* = 8	0.58 × 0.42 × 0.21 mm

### Data collection

**Table:**

Bruker APEXII CCD diffractometer	3132 independent reflections
Radiation source: fine-focus sealed tube	2637 reflections with *I* > 2σ(*I*)
Graphite monochromator	*R*_int_ = 0.014
φ and ω scans	θ_max_ = 28.3°, θ_min_ = 2.8°
Absorption correction: multi-scan (*SADABS*; Bruker, 2008)	*h* = −39→39
*T*_min_ = 0.850, *T*_max_ = 0.943	*k* = −14→13
11843 measured reflections	*l* = −10→9

### Refinement

**Table:**

Refinement on *F*^2^	Primary atom site location: structure-invariant direct methods
Least-squares matrix: full	Secondary atom site location: difference Fourier map
*R*[*F*^2^ > 2σ(*F*^2^)] = 0.037	Hydrogen site location: inferred from neighbouring sites
*wR*(*F*^2^) = 0.103	H-atom parameters constrained
*S* = 1.03	*w* = 1/[σ^2^(*F*_o_^2^) + (0.0463*P*)^2^ + 1.7436*P*] where *P* = (*F*_o_^2^ + 2*F*_c_^2^)/3
3132 reflections	(Δ/σ)_max_ < 0.001
163 parameters	Δρ_max_ = 0.31 e Å^−^^3^
0 restraints	Δρ_min_ = −0.23 e Å^−^^3^

### Fractional atomic coordinates and isotropic or equivalent isotropic displacement parameters (Å^2^)

**Table:**

	*x*	*y*	*z*	*U*_iso_*/*U*_eq_	
Cl1	0.047465 (13)	0.13436 (5)	−0.07319 (6)	0.06357 (16)	
O1	0.30314 (3)	0.00728 (10)	0.48581 (13)	0.0473 (3)	
N1	0.25598 (4)	−0.00421 (12)	0.40628 (16)	0.0450 (3)	
C1	0.24324 (4)	0.09115 (11)	0.31161 (15)	0.0300 (2)	
C2	0.28171 (4)	0.18052 (11)	0.30834 (17)	0.0349 (3)	
H2A	0.2724	0.2680	0.3214	0.042*	
H2B	0.2931	0.1724	0.2029	0.042*	
C3	0.31733 (5)	0.13540 (12)	0.46363 (16)	0.0373 (3)	
H3	0.3141	0.1857	0.5645	0.045*	
C11	0.19537 (4)	0.10199 (11)	0.21455 (15)	0.0295 (2)	
C12	0.18093 (4)	0.20517 (12)	0.11130 (16)	0.0346 (3)	
H12	0.2025	0.2686	0.1017	0.042*	
C13	0.13540 (5)	0.21625 (13)	0.02230 (17)	0.0405 (3)	
H13	0.1256	0.2869	−0.0475	0.049*	
C14	0.10459 (4)	0.12302 (13)	0.03681 (17)	0.0389 (3)	
C15	0.11790 (4)	0.01975 (13)	0.13904 (17)	0.0388 (3)	
H15	0.0962	−0.0432	0.1484	0.047*	
C16	0.16323 (4)	0.00951 (12)	0.22721 (17)	0.0354 (3)	
H16	0.1727	−0.0612	0.2973	0.043*	
C21	0.36712 (4)	0.13532 (11)	0.45344 (15)	0.0321 (3)	
C22	0.39722 (5)	0.22193 (12)	0.54584 (18)	0.0409 (3)	
H22	0.3864	0.2811	0.6151	0.049*	
C23	0.44339 (5)	0.22225 (14)	0.5373 (2)	0.0504 (4)	
H23	0.4639	0.2825	0.5993	0.060*	
C24	0.45938 (5)	0.13514 (15)	0.4389 (2)	0.0491 (4)	
H24	0.4909	0.1347	0.4339	0.059*	
C25	0.42940 (5)	0.04860 (15)	0.3476 (2)	0.0480 (3)	
H25	0.4404	−0.0113	0.2797	0.058*	
C26	0.38362 (5)	0.04870 (13)	0.35468 (17)	0.0399 (3)	
H26	0.3632	−0.0111	0.2913	0.048*	

### Atomic displacement parameters (Å^2^)

**Table:**

	*U*^11^	*U*^22^	*U*^33^	*U*^12^	*U*^13^	*U*^23^
Cl1	0.03330 (19)	0.0913 (3)	0.0612 (3)	0.01429 (18)	0.00056 (16)	0.0042 (2)
O1	0.0324 (5)	0.0532 (6)	0.0521 (6)	−0.0033 (4)	0.0008 (4)	0.0226 (5)
N1	0.0310 (6)	0.0492 (7)	0.0523 (7)	−0.0030 (5)	0.0042 (5)	0.0185 (6)
C1	0.0321 (6)	0.0295 (5)	0.0294 (5)	0.0008 (4)	0.0095 (4)	0.0000 (4)
C2	0.0312 (6)	0.0296 (6)	0.0435 (7)	0.0008 (5)	0.0074 (5)	0.0034 (5)
C3	0.0353 (6)	0.0426 (7)	0.0338 (6)	−0.0007 (5)	0.0076 (5)	−0.0058 (5)
C11	0.0320 (5)	0.0282 (5)	0.0294 (5)	0.0029 (4)	0.0093 (4)	−0.0022 (4)
C12	0.0383 (6)	0.0325 (6)	0.0343 (6)	0.0027 (5)	0.0108 (5)	0.0022 (5)
C13	0.0434 (7)	0.0421 (7)	0.0359 (6)	0.0129 (6)	0.0088 (5)	0.0065 (5)
C14	0.0304 (6)	0.0504 (7)	0.0355 (6)	0.0099 (5)	0.0066 (5)	−0.0044 (6)
C15	0.0326 (6)	0.0397 (7)	0.0449 (7)	−0.0007 (5)	0.0106 (5)	−0.0045 (5)
C16	0.0347 (6)	0.0306 (6)	0.0413 (6)	0.0023 (5)	0.0092 (5)	0.0028 (5)
C21	0.0326 (6)	0.0332 (6)	0.0290 (5)	−0.0006 (4)	0.0036 (4)	0.0011 (5)
C22	0.0439 (7)	0.0338 (6)	0.0428 (7)	−0.0031 (5)	0.0054 (5)	−0.0050 (5)
C23	0.0421 (8)	0.0454 (8)	0.0583 (9)	−0.0138 (6)	0.0002 (6)	0.0011 (7)
C24	0.0320 (7)	0.0534 (9)	0.0615 (9)	0.0014 (6)	0.0099 (6)	0.0110 (7)
C25	0.0443 (8)	0.0490 (8)	0.0529 (8)	0.0073 (6)	0.0160 (6)	−0.0014 (7)
C26	0.0385 (7)	0.0413 (7)	0.0390 (7)	−0.0019 (5)	0.0069 (5)	−0.0068 (6)

### Geometric parameters (Å, º)

**Table:**

Cl1—C14	1.7372 (14)	C13—H13	0.9500
O1—N1	1.4121 (14)	C14—C15	1.385 (2)
O1—C3	1.4597 (18)	C15—C16	1.3816 (18)
N1—C1	1.2818 (17)	C15—H15	0.9500
C1—C11	1.4688 (16)	C16—H16	0.9500
C1—C2	1.4985 (17)	C21—C26	1.3853 (19)
C2—C3	1.5277 (19)	C21—C22	1.3859 (18)
C2—H2A	0.9900	C22—C23	1.393 (2)
C2—H2B	0.9900	C22—H22	0.9500
C3—C21	1.5043 (18)	C23—C24	1.380 (2)
C3—H3	1.0000	C23—H23	0.9500
C11—C12	1.3936 (17)	C24—C25	1.380 (2)
C11—C16	1.3979 (18)	C24—H24	0.9500
C12—C13	1.3893 (18)	C25—C26	1.378 (2)
C12—H12	0.9500	C25—H25	0.9500
C13—C14	1.380 (2)	C26—H26	0.9500
			
N1—O1—C3	108.20 (9)	C13—C14—Cl1	119.96 (11)
C1—N1—O1	109.39 (10)	C15—C14—Cl1	118.45 (11)
N1—C1—C11	120.17 (11)	C16—C15—C14	119.06 (12)
N1—C1—C2	113.33 (11)	C16—C15—H15	120.5
C11—C1—C2	126.46 (10)	C14—C15—H15	120.5
C1—C2—C3	100.08 (10)	C15—C16—C11	120.79 (12)
C1—C2—H2A	111.8	C15—C16—H16	119.6
C3—C2—H2A	111.8	C11—C16—H16	119.6
C1—C2—H2B	111.8	C26—C21—C22	119.26 (12)
C3—C2—H2B	111.8	C26—C21—C3	121.01 (11)
H2A—C2—H2B	109.5	C22—C21—C3	119.73 (12)
O1—C3—C21	108.85 (10)	C21—C22—C23	120.04 (13)
O1—C3—C2	103.42 (10)	C21—C22—H22	120.0
C21—C3—C2	117.74 (11)	C23—C22—H22	120.0
O1—C3—H3	108.8	C24—C23—C22	120.07 (13)
C21—C3—H3	108.8	C24—C23—H23	120.0
C2—C3—H3	108.8	C22—C23—H23	120.0
C12—C11—C16	118.86 (11)	C23—C24—C25	119.78 (14)
C12—C11—C1	120.90 (11)	C23—C24—H24	120.1
C16—C11—C1	120.22 (11)	C25—C24—H24	120.1
C13—C12—C11	120.73 (12)	C26—C25—C24	120.30 (14)
C13—C12—H12	119.6	C26—C25—H25	119.8
C11—C12—H12	119.6	C24—C25—H25	119.8
C14—C13—C12	118.98 (12)	C25—C26—C21	120.55 (13)
C14—C13—H13	120.5	C25—C26—H26	119.7
C12—C13—H13	120.5	C21—C26—H26	119.7
C13—C14—C15	121.58 (12)		
			
C3—O1—N1—C1	13.27 (15)	C13—C14—C15—C16	−0.6 (2)
O1—N1—C1—C11	−179.87 (10)	Cl1—C14—C15—C16	179.98 (10)
O1—N1—C1—C2	2.47 (16)	C14—C15—C16—C11	0.26 (19)
N1—C1—C2—C3	−15.95 (14)	C12—C11—C16—C15	0.04 (18)
C11—C1—C2—C3	166.57 (11)	C1—C11—C16—C15	178.79 (11)
N1—O1—C3—C21	−148.44 (11)	O1—C3—C21—C26	44.82 (16)
N1—O1—C3—C2	−22.50 (13)	C2—C3—C21—C26	−72.34 (16)
C1—C2—C3—O1	21.97 (12)	O1—C3—C21—C22	−134.27 (12)
C1—C2—C3—C21	142.02 (11)	C2—C3—C21—C22	108.56 (14)
N1—C1—C11—C12	179.87 (12)	C26—C21—C22—C23	0.8 (2)
C2—C1—C11—C12	−2.81 (18)	C3—C21—C22—C23	179.87 (12)
N1—C1—C11—C16	1.15 (18)	C21—C22—C23—C24	−1.0 (2)
C2—C1—C11—C16	178.47 (12)	C22—C23—C24—C25	0.7 (2)
C16—C11—C12—C13	0.01 (18)	C23—C24—C25—C26	−0.1 (2)
C1—C11—C12—C13	−178.73 (11)	C24—C25—C26—C21	−0.1 (2)
C11—C12—C13—C14	−0.36 (19)	C22—C21—C26—C25	−0.2 (2)
C12—C13—C14—C15	0.7 (2)	C3—C21—C26—C25	−179.31 (13)
C12—C13—C14—Cl1	−179.94 (10)		

### Hydrogen-bond geometry (Å, º)

Cg is the centroid of the C11–C16 ring.

**Table:**

*D*—H···*A*	*D*—H	H···*A*	*D*···*A*	*D*—H···*A*
C12—H12···N1^i^	0.95	2.74	3.657 (2)	163
C12—H12···O1^i^	0.95	2.65	3.390 (2)	135
C2—H2*B*···O1^ii^	0.99	2.67	3.466 (2)	138
C26—H26···O1^ii^	0.95	2.70	3.431 (2)	134
C22—H22···*Cg*^iii^	0.95	2.81	3.721 (3)	162

Symmetry codes: (i) −*x*+1/2, *y*+1/2, −*z*+1/2; (ii) *x*, −*y*, *z*−1/2; (iii) −*x*+1/2, −*y*+1/2, −*z*+1.
